# Protein acetylation and aging

**DOI:** 10.18632/aging.100398

**Published:** 2011-10-31

**Authors:** Jin-Ying Lu, Yu-Yi Lin, Heng Zhu, Lee-Ming Chuang, Jef D. Boeke

**Affiliations:** ^1^ Department of Laboratory Medicine, National Taiwan University Hospital; ^2^ Department of Internal Medicine, National Taiwan University Hospital; ^3^ Department of Oncology, National Taiwan University Hospital; ^4^ Institute of Biochemistry and Molecular Biology, College of Medicine, National Taiwan University, Taipei 100, Taiwan; ^5^ Department of Pharmacology and Molecular Sciences, Johns Hopkins University School of Medicine; ^6^ Departments of Molecular Biology and Genetics, Johns Hopkins University School of Medicine; ^7^ The High Throughput Biology Center, Johns Hopkins University School of Medicine, Baltimore, MD 21205, USA

Aging is now viewed as a plastic phenotype that can be altered by nutritional, pharmacological and genetic manipulations. However, most pro-longevity mutations are discovered by systematic gene deletion or RNA interference screens, which mainly reveal abolished or diminished gene functions [1]. In our recent publications [2, 3], we used global acetylation proteome screens to study aging in yeast, and showed that enhancing the function of certain genes through specific acetylation can promote longevity. The genes involved are by no means strangers – acetylation of yeast AMPK regulatory subunit by the essential lysine acetyl-transferase (KAT) complex NuA4 leads to increased protein-protein interaction and extension of lifespan.

It is well known that acetylation of histone proteins in cultured human fibroblasts decreases during aging, which is believed to be directly related to decreased metabolic rate and reproductive capacity associated with aging [4]. However, histone deacetylation is not likely to be a universal driving force of aging because histone acetylation and deacetylation mimetics similarly shortened life span [5], which could simply reflect nonspecific fitness decreases in both instances. Extension of lifespan promoted by certain genetic and/or pharmacological perturbations will more likely lead to identification of bona fide regulatory factors of aging.

Starting from a proteome-wide search for non-histone substrates of NuA4 complex, we unexpectedly found Sip2, one of the three regulatory β subunit of yeast AMPK, could be acetylated and deacetylated by NuA4 and Rpd3, respectively. Sip2 acetylation enhances its interaction with the yeast AMPK catalytic subunit, Snf1, and inhibits its kinase activity. Interestingly, Sip2 acetylation declined significantly with yeast replicative aging. Restoration of Sip2 acetylation by deleting the deacetylase Rpd3 or through use of acetylation mimetics both slowed cellular growth, increased resistance to oxidative damage, and increased lifespan. These pro-longevity effects result from reduced AMPK catalytic activity and subsequent activation of downstream target Sch9 [2].

**Figure 1:**
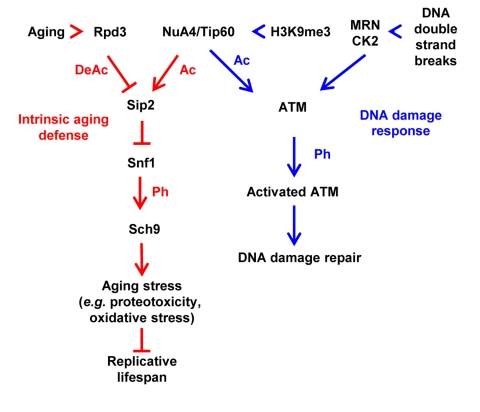
Mixed acetylation phosphorylation cascades of intrinsic aging defense (shown in red) and DNA damage response pathways (shown in blue). With aging, NuA4 acetylation of Sip2 declines, resulting in Snf1 activation and subsequent Sch9 phosphorylation. Forced Sip2 acetylation eliminates Snf1 activation and Sch9 phosphorylation, delays the aging process and leads to life span extension. Similarly, following occurrence of double strand breaks (DSBs), MRN complex and CK2 are recruited to the DSB site, whereCK2 leads to the release of inhibition from tri-methylated H3K9 (H3K9me3) and the recruitment of Tip60/ATM complex to MRN. MRN activates Tip60 acetyltransferase activity and in turn results in the acetylation and subsequent phosphorylation and activation of ATM. Ac, acetylation; Ph, phosphorylation.

We note that a similar mixed acetyltransferase kinase cascade exists in mammalian cells, with the NuA4 homolog Tip60 acetyltransferase complex at the “top”, possibly counteracted by a deacetylase, in which the acetylation signal is transmitted to a cascade of two kinases, ATM/ATR - CHK1/CHK2,to execute a crucial DNA damage response [6]. Thus this may represent a common motif in eukaryotic regulatory networks that was not previously appreciated (Figure 1).

Aging is conventionally thought to be characterized by accumulation of molecular, cellular, and organ damage, leading to increased vulnerability to disease and death [7]. Our data, on the contrary, support the idea that the gradual loss of a crucial component promoting “healthy young status” might underlie an intrinsic aging process. Many of the mutations that extend life span decrease the activity of external nutrient signaling, such as the IGF (insulin-like growth factor)/insulin and the TOR (target of rapamycin) pathways, suggesting that they may induce a metabolic state similar to that resulting from periods of food shortage. However, dietary restriction can also have destructive effects, impairing critical functions such as immunity, causing susceptibility to infections [7]. Our results suggest the possible benefit of manipulating an intrinsic aging pathway that is independent of nutrition availability, a potential therapeutic route that might be able to bypass shortcomings of calorie restriction.

Fundamental biological processes are usually conserved across species. Many of mechanisms regulating longevity are no exception, especially those involved in controlling cellular metabolism, nutrition sensing and stress response. Although the relationship of our yeast research to human systems is still uncertain, we are enthusiastic to explore this newly identified aging mechanism to higher organisms. Thinking ahead, one can imagine that by restoring the distorted homeostasis associated with aging one could ultimately improve the welfare and quality of life of aged humans.
